# In the Thick of Air Pollution: Particles May Contribute to Atherosclerosis

**Published:** 2005-02

**Authors:** Bob Weinhold

Fine particles damage more than just the lungs—the effects reach all the way to the inner lining of the arteries, report researchers from the University of Southern California’s Keck School of Medicine, led by Nino Künzli **[*EHP* 113:201–206]**. Their paper provides the first epidemiologic evidence linking atherosclerosis with air pollution. It also adds to the growing body of evidence linking pollutants with cardiovascular damage of many types.

To uncover this new evidence, Künzli and colleagues studied 798 men and women from the Los Angeles area. These study subjects were already participating in two clinical trials investigating other aspects of atherosclerosis, or thickening and hardening of the arteries. The selected participants were generally healthy people over 40 who showed some signs of increased risk of cardiovascular disease.

To gauge the participants’ exposure to fine particles, the researchers began with year 2000 data from 23 monitoring stations in the Los Angeles basin, then interpolated the data, using a geographic information system and geostatistics, to assign long-term mean ambient particulate matter concentrations to each participant’s address. According to the authors, fine particle concentrations have changed little over the past 5–10 years, and the 2000 data are representative of long-term exposure. This novel approach allowed a more accurate approximation of exposures than simply using readings from the closest monitor.

The potential effects of fine particles were assessed using data gathered previously on the thickness of the two inner layers of the carotid artery, which feeds the head and neck. This technique has become generally accepted as a reliable indicator of atherosclerosis, which happens slowly in all people and is a leading contributor to death in most countries. In their statistical analysis of the data the researchers accounted for other factors such as diet, use of vitamin supplements and hormone replacement drugs, physical activity, blood pressure, education, and income.

Overall, the researchers found that for each increase in fine particles of 10 micrograms per cubic meter (μg/m^3^), the two inner layers of the carotid artery thickened by about 4%. For the selected monitoring stations, fine particle concentrations ranged from 5.2 to 26.9 μg/m^3^. That means the most-exposed participants in this study experienced about 8% more artery thickening than the least-exposed participants.

However, not all participants were found to be equally vulnerable. Women over age 60 experienced artery thickening of about 15% for each 10-μg/m^3^ increase. In general women were much more vulnerable than men, although no link was observed for women taking hormone replacement drugs. Others who were significantly more vulnerable included both male and female nonsmokers as well as men and women taking drugs to reduce cholesterol.

The researchers studied one other pollutant, ozone, and found some correlations but no significant link with atherosclerosis. However, they acknowledge some weaknesses in their ability to detect links, given their methods and available data. For example, previous studies cited by the authors indicate that outdoor ozone measurements are very weakly correlated with actual personal exposures.

Künzli and colleagues acknowledge other limitations as well, and say much more research needs to be done. For instance, the selection of participants focused on a relatively healthy population and may have excluded those at highest risk. Also, the relatively low numbers of people in each subgroup analyzed add to the possibility that the findings for these groups may not be accurate.

In addition, the projections for exposure to fine particulates measured outdoors have some small margin of error. Using just these data also excludes exposures in vehicles, workplaces, and other enclosed settings, where people generally spend the vast majority of their time. Other studies have found significant increases in indoor and in-vehicle concentrations of many pollutants, including fine particles, compared to measurements taken at nearby outdoor monitoring stations.

## Figures and Tables

**Figure:**
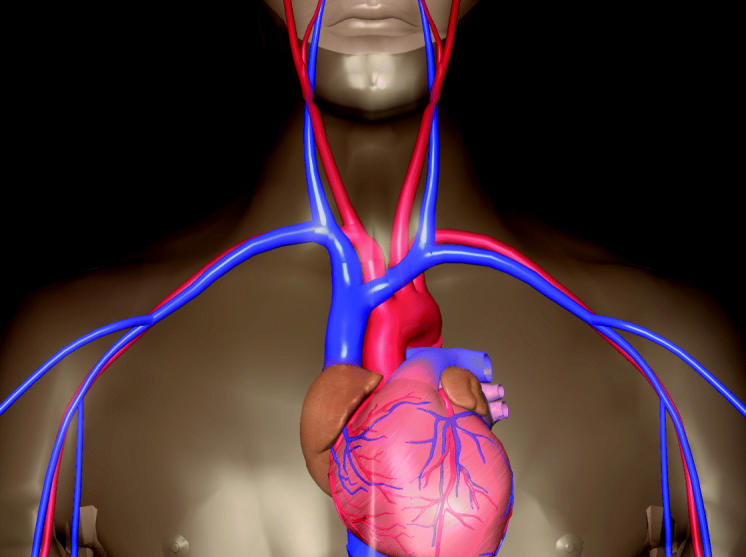
**In the thick of it.** New data provide the first epidemiological evidence that exposure to fine particles can contribute to atherosclerosis—a thickening and hardening of the arteries—in generally healthy adults.

